# hiPSC-Derived Cells as Models for Drug Discovery 2.0

**DOI:** 10.3390/ijms24065727

**Published:** 2023-03-17

**Authors:** Rivka Ofir

**Affiliations:** 1 The Regenerative Medicine and Stem Cell (RMSC) Research Center, Ben-Gurion University of the Negev, Beer Sheva 84105, Israel; rivir@bgu.ac.il; 2 Dead Sea & Arava Science Center, Masada 8691000, Israel

Human-induced pluripotent stem cells (hiPSCs) serve as a sustainable resource for studying the molecular foundation of disease development, including initiation and deterioration. Although the process of reprogramming adult cells is accompanied by the obliteration of part of the epigenetic signature, usually upon differentiation of hiPSCs into specific cells, such as brain, heart, or muscle cells, these cells are adequate models for studying disease pathology and for drug discovery, as described in this Special Issue. 

In considering iPSCs as models for mono/complex diseases and as a potential future replacement for animal studies, Costa et al. [1] suggest an original method to compensate for the immature electrophysiology of human-induced pluripotent-stem-cell-derived cardiomyocytes via co-culturing them with a human embryonic kidney (HEK) cell line expressing the Inward Rectifier Channel Kir2.1 (HEK-IK1). They tested the potential of this co-culture system to generate an electrical syncytium with an adult-like cardiac electrophysiology and concluded that it can be achieved.

Alvarez et al. [2] observed an increase in the expression of cardiac damage markers and cardiac channels, an increase in oxidative stress, a decrease in mitochondrial respiration and autophagy, and lipid accumulation in hiPSC-derived cardiomyocytes containing a mutation in the *PCCB* genes (encoding the two subunits of the PCC enzyme, which carboxylates propionyl-CoA to D-methylmalonyl-CoA; this is known as propionic acidemia disorder). They suggest that the upregulation of cardiac-enriched miRNAs can explain these changes and that they can serve as new therapeutic targets for intervention strategies for this cardiomyopathy-associated disorder. 

Interestingly, in a study of an hiPSC line generated from a patient with mutations in a factor related to autophagy (pleckstrin homology; additionally, RUN-Domain-Containing M2 (PLEKHM2)), Ben-Zvi et al. [3] present impaired functions of hiPSC-derived motor neurons (iMNs); electrophysiological measurements revealed that PLEKHM2[delAG] iMN cultures displayed delayed functional maturation and more frequent and unsynchronized activity. These impaired electrical functions were associated with an enlarged lysosome. These results are of great interest as autophagic dysregulation is associated with the pathogenesis of neurodegenerative diseases, and modulation of autophagy holds considerable potential as a therapeutic target for such disorders. Recently, a study using the same iPSC line identified an association of a PLEKHM2 mutation with cardiomyopathy and left ventricular non-compaction (DCM-LVNC), further supporting the importance of autophagy for normal cardiac function [4]. 

Walker et al. [5] establish a platform based on hiPSCs for screening compounds for cardiac developmental toxicity as a relevant alternative to mouse stem cells. They describe differences in the contraction incidence of iPSC-derived cardiomyocytes for cultures treated with 5-flurorouracil (5-FU) and all-trans retinoic acid (atRA) compared to the untreated control. In this platform, TBX5 mRNA expression yielded accurate early toxicity classifications, as expected from murine embryonic stem cells assays. When conventional cigarette smoke (Marlboro Red 100) and Snus smokeless tobacco (Camel Snus) were tested, dose-dependent reductions in the formation of active contractile clusters and structures were monitored for both tobacco products. The reuslts of this study suggest that hiPSC-derived cardiomyocytes can effectively be used to replace traditional animal embryotoxicity screens. This result opens opportunities for high-throughput screening using automated assessments of early tissue marker endpoints as outlined, reducing culture time and the use of animals.

The review by Gabriele Bonaventura et al. [6] reports on the use of iPSCs as a disease model for drug development in the context of neurological disorders, including Alzheimer’s (AD) and Parkinson’s disease (PD), amyotrophic lateral Sclerosis (ALS), and fragile X syndrome (FRAX).

Kutschenko et al. [7] investigated the molecular and functional phenotype of striatal medium spiny neurons (MSNs) differentiated from myoclonus–dystonia (DYT-SGCE) patient-derived induced pluripotent stem cells (iPSCs) via calcium imaging, whole-cell patch clamp recordings, the gene expression of voltage-gated Ca^2+^ channel subunits, and ionotropic receptor subunits and the density of GABAergic synapses. The results indicated elevated basal intracellular Ca^2+^ levels and lower frequency of spontaneous Ca^2+^ signals, elevated Ca^2+^ amplitudes upon glycine and acetylcholine application, and larger miniature postsynaptic current (mPSC) amplitudes in SGCE MSNs as compared to healthy MSNs. The contribution of this in vitro model of DYT-SGCE myoclonus–dystonia to the understanding of the functional phenotype and pathophysiology of the disease may help to advance the development of new therapeutic strategies. 

The study by Tate et al. [8] sheds light on the effects of in utero exposure of embryos to environmental factors such as pharmaceuticals (e.g., selective serotonin reuptake inhibitors, fluoxetine) that may alter synapse formation and increase risk of neurodevelopmental abnormalities. They used cortical spheroids that express serotonin transporter, a system that recapitulates the early developmental expression of serotonin transporter associated with cortical pyramidal neurons. Following treatment with fluxetine, neuronal activity was repressed in a dose-dependent manner. This study provides a foundation for future studies aiming to combine serotonergic innervation with cortical spheroids and assess the influence of fluoxetine-induced alterations in serotonin levels on brain development.

Kuriyama et al. [9] described induced-pluripotent-stem-cell-derived myeloid lines (iPS-MLs) that act as dendritic-cell-like antigen-presenting cells. The 4-1BBL gene (CD137L) was introduced to an iPS-ML to enhance its antigen presentation ability. These cells indeed amplified the antigen-specific T cells and promoted T-cell infiltration into tumor tissues in a mouse melanoma model. These results suggest that these iPS-ML cells expressing 4-1BBL may be a candidate for future immune cell therapy aiming to turn an immunologically “cold tumor” into a “hot tumor”.

In their review, Heider et al. [10] describe how human-iPSC-derived glia cells can serve as a model for neuropsychiatric research (such as schizophrenia or autism spectrum disorder) and for drug development. The review emphasizes the evidence that aberrant neuroinflammatory responses of glial cells account for synaptic pathologies through deregulated communication and reciprocal modulation. As such, microglia and astrocytes emerge as central targets for anti-inflammatory treatment to preserve the organization and homeostasis of the central nervous system. Two- and three-dimensional co-culture models are suggested as efficient in vitro models for drug development.

Abati et al. [11] reviews the development of in vitro iPSC-derived muscle models for muscular dystrophies. These models recapitulate these diseases, e.g., lack of myofiber proteins as well as other specific pathological hallmarks, such as inflammation, fibrosis, and reduced muscle regenerative potential. The review describes how these platforms have been used to assess genetic correction strategies, such as gene silencing, gene transfer, and genome editing, with clustered regularly interspaced short palindromic repeats (CRISPRs)/CRISPR-associated protein 9 (Cas9), as well as to evaluate novel small molecules aimed at ameliorating muscle degeneration. 

Methods: this Special Issue presents several articles describing modified protocols for the efficient generation of specific iPSC-derived cellular models. 

Baldassari et al. [12] aimed to develop feeder-free conditions for generating functional glutamatergic neurons suitable for future clinical applications. By including specific neurotrophins in a Geltrex-coated substrate, the hiPSC-derived neurons acquired Ca^2+^-dependent glutamate release properties as a hallmark of neuronal maturation. 

Lanfer et al. [13] describe an efficient and reproducible protocol to convert iPSCs into functional induced microglia-like cells (iMGLs). In the first step, iPSCs are differentiated into hematopoietic progenitor cells (HPCs), and in the second step, HPCs mature to iMGLs that respond to the inflammatory stimuli LPS.

Johnson Chacko et al. [14] describe the role of the extracellular matrix and integrin receptor genes in human iPSC-derived otic neurosensory differentiation. They identify the critical role of novel gene encoding of the extracellular matrix and integrin receptor interactions with otic neurosensory lineage genes in early neurosensory development and cell fate determination in the human fetal inner ear.

Helmi et al. [12] show that Notch inhibition enhances the differentiation of both mouse embryonic and induced pluripotent stem cells. iPSC differentiation towards bone formation is an important model for studying bone healing. The osteogenic differentiation of bone progenitor cells and bone marrow mesenchymal stem cells (MSCs) depends on the interaction between different types of cell signaling, including Notch signaling; the DAPT γ-secretase inhibitor of the Notch signaling pathway enhances osteogenic differentiation. 

The review by Matsumoto et al. [15] describes how iPSC-derived complex organs serve as faithful models when intra- and inter-organ interactions play an essential role. They describe how three-dimensional organoids, bioengineering, and organ-on-a-chip technology have great potential for constructing multicellular tissues, generating the functional organs from hPSCs, and recapitulating complex tissue functions for better biological research and disease modeling.

The review by Bigarreau et al. [16] discusses the generation of relevant models for neurological disorders and their importance for both target identification and drug discovery. These models are relevant for a broad range of neurodegenerative and neurodevelopmental disorders. The review provides an overview of the generation and modeling of both neuronal and glial cells from human iPSCs and includes a brief synthesis of recent work investigating neuroglial interactions using hiPSCs in a pathophysiological context.

The review by Antonov et al. [17] describes the current status of iPSC-derived dopamine neurons for screening drugs against Parkinson’s disease (PD). Although 3D culture methods closely mimic the brain microenvironment, the models are still not quite applicable; better differentiation protocols are required. To close the current knowledge gap, the review suggests several ways in which the use of iPSC-derived neurons for PD drug development can be improved.

Pasqua et al. [18] describe iPSC-derived hepatobiliary lineages (hepatocytes and cholangiocytes) as models for drug discovery. The review emphasizes the importance of adopting three-dimensional (3D) cell culture systems and tissue engineering approaches to build functional tissues that will mature towards hepatobiliary lineages.
